# Human Fibroblast Gene Expression Modulation Using 940 NM Diode Laser

**DOI:** 10.1038/s41598-019-48595-2

**Published:** 2019-08-19

**Authors:** Rebeca Illescas-Montes, Lucía Melguizo-Rodríguez, Olga García-Martínez, Elvira de Luna-Bertos, Francisco Javier Manzano-Moreno, Concepción Ruiz, Javier Ramos-Torrecillas

**Affiliations:** 1 Biomedical Group (BIO277), Department of Nursing, Faculty of Health Sciences, University of Granada, Granada, Spain; 2 Instituto Investigación Biosanitaria ibs.Granada, ibs.Granada, C/ Doctor Azpitarte 4, 4ª planta, Granada, 18012 Spain; 3 Biomedical Group (BIO277). Department of Stomatology, School of Dentistry, University of Granada, Granada, Spain; 4 Institute of Neuroscience, University of Granada, Centro de Investigación Biomédica (CIBM). Parque de Tecnológico de la Salud (PTS), Granada, Spain

**Keywords:** Therapeutics, Skin diseases, Growth factor signalling

## Abstract

Low-Level Laser Therapy is used as regenerative therapy in different clinical fields. This is due to its photobiomodulation effect *via* cell signaling on different cell populations, Including fibroblasts, cells involved in tissue regeneration and healing. The aim was to analyze the effect of 940 nm diode laser on the gene expression of different markers involved in fibroblast growth, differentiation, and migration. Real-time polymerase chain reaction (q-RT-PCR) was used to quantify the expression of fibroblast growth factor (FGF), connective tissue growth factor (CTGF), vascular-endothelial growth factor (VEGF), transforming growth factor β1 (TGF-β1), TGFβ-receptors (TGFβR1, TGFβR2, and TGFβR3), discoidin-domain receptor-2 (DDR2), matrix metalloproteinase-2 (MMP2), α-actin, fibronectin, decorin, and elastin on human fibroblast, treated with single dose (T1) or two doses (T2) of diode laser at 0.5 Watts and 4 J/cm^2^. A significant increase in the expression of FGF, TGF-β1, TGFβR1, TGFβR2, α-actin, fibronectin, decorin, DDR2 and MMP2 was observed after both treatments. A decrease was observed in expression of elastin (T1 and T2), and CTGF (T2). These changes underlie the biostimulatory effect of laser on fibroblasts, which translates into an increase in short-term proliferation and in long-term differentiation to myofibroblasts. These data support the therapeutic potential of diode laser for wound repair.

## Introduction

Fibroblasts are responsible for forming and maintaining soft connective tissue and constitute the main source of collagen for the extracellular matrix (ECM). Fibroblasts are in a quiescent state in healthy conjunctive tissue, being metabolically active but unable to proliferate, although they slowly synthetize, degrade, and organize the ECM to maintain the tissue structure^1,2^. However, tissue injuries induce major changes in cell signaling that translate into cell activation, stimulating the formation of granulation tissue with a high cell component (fibroblasts, macrophages, myofibroblasts, neovasculature) and thereby contributing to the creation of new mature connective tissue, favoring tissue regeneration^1^. Highly active ECM formation/synthesis by fibroblasts exceeds degradation in this phase^3^. However, processes of connective tissue re-epithelialization, healing, and angiogenesis can be compromised in cases of chronic injury^4^.

Low-Level Laser Therapy (LLLT) is applied in multiple clinical fields^5^. It is based on the application of low-power amplified light radiation capable of promoting biochemical, bioelectric, and structural changes that produce analgesic, anti-inflammatory, and/or microcirculation-stimulating effects^6–8^. Its usefulness in the treatment of chronic wounds is currently under debate^8,9^.

The photobiomodulation effect of lasers depend on their type and the emission wavelength and energy selected^8^. The 940 nm diode laser has biostimulatory effect on various cell populations involved in regenerative processes, including osteoblasts in hard tissues^10–12^ and fibroblasts in soft tissues^13^. Treatment with this laser for 24 or 72 h increased the proliferative capacity of cultured human fibroblasts as a function of the energy dose applied and induced their expression of α-actin, a marker of fibroblast differentiation^13^.

The objective of this study was to determine the effects on fibroblasts of treatment with 940 nm diode laser, analyzing the expression of fibroblast growth and differentiation markers and exploring the usefulness of LLLT in soft connective tissue healing.

## Results

**Figure 1:**
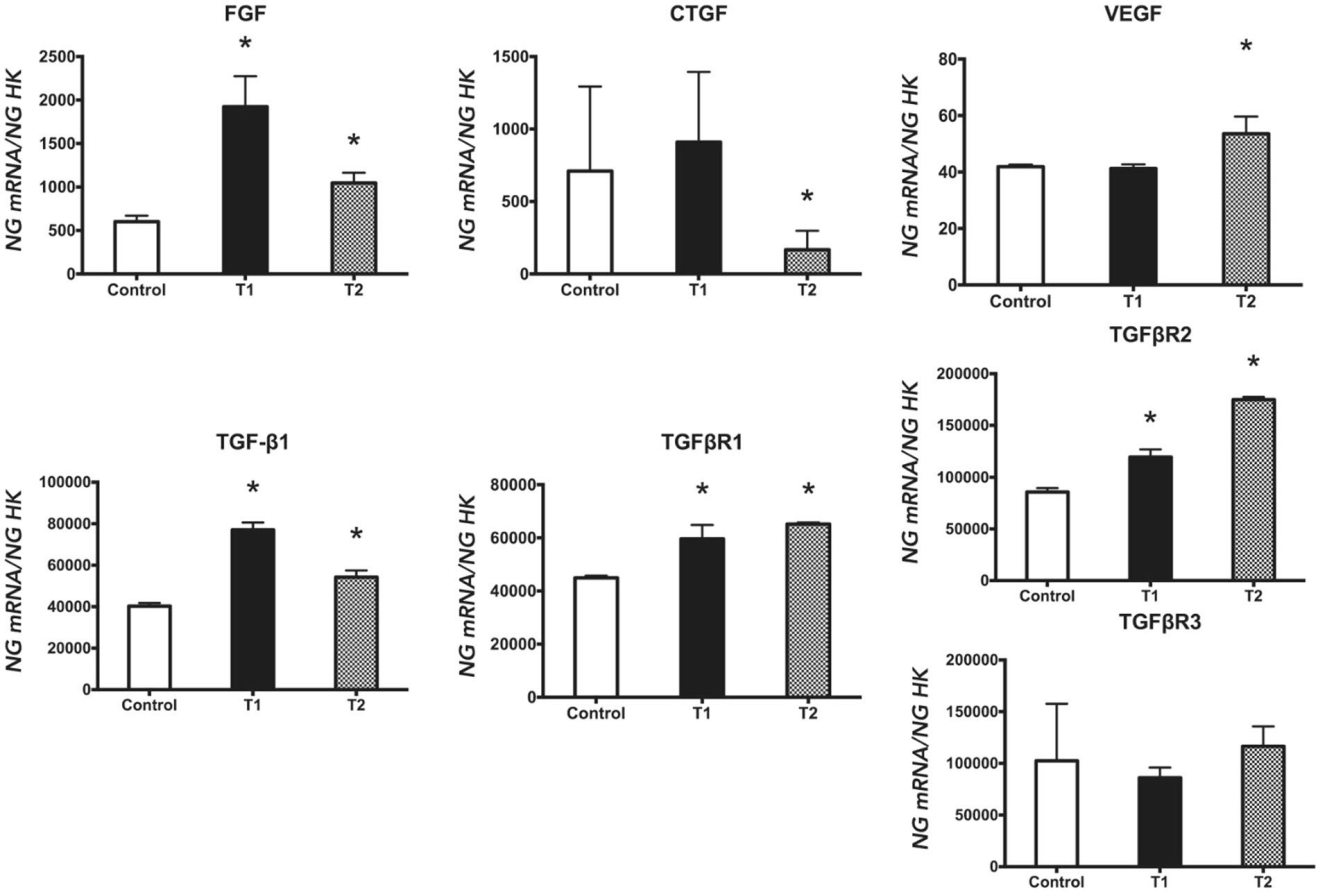
Expression of fibroblasts genes (FGF, CTGF, VEGF, TGF-β1, TGFβR1, TGFβR2, and TGFβR3) treated with a 940 nm diode laser at 0.5 Watts and 4 J/cm^2^, following two treatment protocols: one dose (T1) and two doses (T2). Data are expressed as ng of mRNA per average ng of housekeeping mRNAs ± SD. *p ≤ 0.05 Significance in the treated group was determined with respect to the control group.

**Figure 2:**
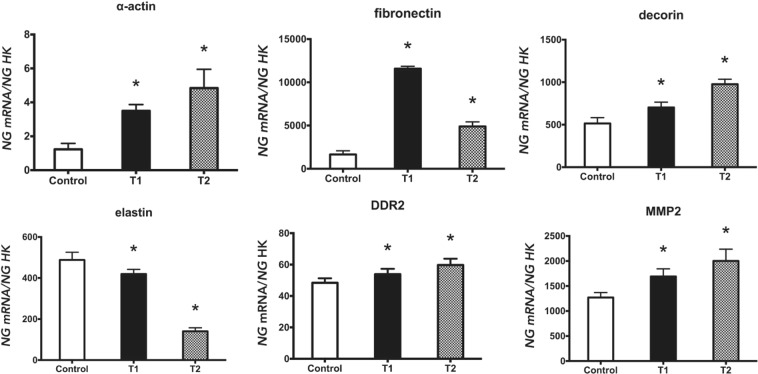
Expression of fibroblasts genes (α-actin, fibronectin, decorin, elastin, DDR2, and MMP2) treated with a 940 nm diode laser at 0.5 Watts and 4 J/cm^2^, following two treatment protocols: one dose (T1) and two doses (T2). Data are expressed as ng of mRNA per average ng of housekeeping mRNAs ± SD. *p ≤ 0.05 Significance in the treated group was determined with respect to the control group.

The gene expression of growth factors is depicted in Fig. 1, whereas the gene expression of differentiation markers and extracellular matrix elements is showed in Fig. 2.

### Effect of diode laser on the expression of FGF, CTGF, VEGF, TGF-β1, and TGF-β1 receptors genes

Quantitative RT-PCR (q-RT-PCR) analysis was used to evaluate the expression of the fibroblast markers assayed. As depicted in Fig. 1, cells receiving a single dose (T1) showed increased gene expression of FGF, TGF-β1, TGFβR1, and TGFβR2, while cells receiving two doses (T2) showed increased expression of all markers except for TGFβR3, which did not change, and CTGF, whose expression was significantly reduced with respect to controls.

### Effect of diode laser on the expression of α-actin, fibronectin, decorin, elastin, DDR2, and MMP2

Figure 2 displays the quantification of α-actin, fibronectin, decorin, elastin, DDR2, and MMP2 gene expression by q-RT-PCR. As observed, the expression of α-actin, fibronectin, decorin, DDR2 and MMP-2 was increased *versus* controls with both treatments (T1 and T2), whereas the expression of elastin was reduced with T1 and T2 treatments.

The Table 1 shows the gene expression of the different markers studied and the difference between the T1 and T2 group.

**Table 1:** Gene expression of fibroblasts treated with a 940 nm diode laser at 0.5 Watts and 4 J/cm^2^, following two treatment protocols: one dose (T1) and two doses (T2).

Gen	Treatment	Mean	Standard Deviation	*p value*
FGF	Control	601.45	68.44	
	T1	1924.05	349.99	0.05*
	T2	1046.69	117.88	0.05*
	T1 Vs T2			0.513
CTGF	Control	710.4	582.65	
	T1	909.96	483.99	0.497
	T2	167.73	130.26	0.042*
	T1 Vs T2			0.043*
VEGF	Control	41.9	0.73	
	T1	41.25	1.46	0.658
	T2	53.55	6.1	0.05*
	T1 Vs T2			0.05*
TGF-β1	Control	40272.68	1564.55	
	T1	77088.78	3480.91	0.05*
	T2	54227.56	3255.26	0.05*
	T1 Vs T2			0.275
TGFβR1	Control	44943.09	800.95	
	T1	59638.95	5223.23	0.05*
	T2	65203.3	610.3	0.05*
	T1 Vs T2			0.268
TGFβR2	Control	85792.51	3856.83	
	T1	119296.52	7411.73	0.002*
	T2	174964.17	2677.26	0.000*
	T1 Vs T2			0.05*
TGFβR3	Control	102466.73	55209.72	
	T1	86085.74	9883.43	0.497
	T2	116559.82	19233.12	0.661
	T1 Vs T2			0.014*
α-actin	Control	1.23	0.35	
	T1	3.5	0.37	0.034*
	T2	4.84	1.11	0.021*
	T1 Vs T2			0.034*
fibronectin	Control	1649.99	428.63	
	T1	11576.43	276.38	0.05*
	T2	4893.05	529.88	0.05*
	T1 Vs T2			0.05*
decorin	Control	514.9	67.89	
	T1	702.46	62.83	0.05*
	T2	976.28	59.02	0.05*
	T1 Vs T2			0.001*
elastin	Control	488.14	37.52	
	T1	419.71	22.39	0.05*
	T2	140.3	17.04	0.05*
	T1 Vs T2			0.05*
DDR2	Control	48.39	2.87	
	T1	53.81	3.49	0.05*
	T2	59.72	4.02	0.05*
	T1 Vs T2			0.05*
MMP2	Control	1270.19	99.45	
	T1	1691.09	152.54	0.034*
	T2	2002.29	233.74	0.034*
	T1 Vs T2			0.05*

FGF, fibroblast growth factor; CTGF, connective tissue growth factor; VEGF, vascular endothelial growth factor; TGF-β1, transforming growth factor β1; TGFβR1, transforming growth factor β-receptor 1; TGFβR2, transforming growth factor β-receptor 2; TGFβR3, transforming growth factor β-receptor 3; DDR2, discoidin domain receptor 2; MMP2, matrix metalloproteinase-2. Data are expressed as mean of ng of mRNA per average ng of housekeeping mRNAs and Standard Deviation. *p ≤ 0.05.

## Discussion

Treatment with 940 nm diode laser exerts a photobiomodulation effect on fibroblasts under specific application (energy dose/transmission mode) conditions^13^. The present study demonstrates that *in vitro* treatment with this laser modulates the expression of the human fibroblast markers FGF, CTGF, VEGF, TGF-β1, TGFβR1, TGFβR2, TGFβR3, α-actin, fibronectin, decorin, elastin, DDR2, and MMP2, although the effect depended on the treatment protocol. The proteins encoding these markers play a major role in wound healing by stimulating fibroblast proliferation, migration, and/or maturation^3,14,15^.

FGF promotes fibroblast mitogenesis, favoring the formation of granulation tissue and promoting re-epithelization and damaged tissue remodeling^16^. Both of our treatments with 940 nm diode laser (single dose or two doses) significantly increased FGF expression, although the effect was lower with two doses. Previous studies reported a significance increase in the growth rate of human fibroblasts at 24 h after a single treatment with this laser under the same energy conditions (0.5 W, 4/Jcm^2^) but not at 72 h^13^. Khoo *et al*.^17^ observed a significant increase in FGF expression in fibroblasts from diabetic rats irradiated with diode laser at a single energy dose (810 nm, 10 mW, 1 J/cm^2^). Likewise, Damante *et al*.^18^ also recorded an increase in the FGF expression of cultured gingival fibroblasts treated with infrared laser at different energy densities (780 nm, 40 mW, 3 J/cm^2^, and 5 J/cm^2^).

TGF-β1 is a growth factor with multiple functions, including stimulation of fibroblast proliferation, migration, and adhesion and promotion of the production of ECM elements^19^. TGF-β1 also favors the maturation of fibroblasts, inducing their differentiation to myofibroblasts, which are responsible for contraction and for synthetizing ECM elements^20^. Nonetheless, TGF-β1 overexpression has been considered as largely responsible for hypertrophic scars and keloids because of its stimulation of CTGF secretion^21^. In the present study, both laser treatments (single dose and two doses) significantly increased expression by the fibroblasts of TGF-β1 and its receptors TGFβR1 and TGFβR2. The increase in TGF-β1 expression was lower after two doses than after one, which may be explained by the maturation process of the fibroblasts, given that TGF-β1 expression induced by the single dose may be related to a fibroblastic cell phenotype, while the presence of differentiated myofibroblasts would have reduced this expression at 72 h after the second dose. CTGF expression was not changed by the single-dose treatment but was significantly decreased by the two-dose treatment.

The proteoglycan decorin has key functions in the ECM, inactivating TGF-β and CTGF. For this reason, decorin levels are lower and elastin fiber deposits higher in hypertrophic scars than in normal skin^22,23^. Both laser treatments produced a significant increase *versus* controls in the fibroblast expression of decorin that was higher in the fibroblasts receiving two doses, which would be related to the expression pattern of TGF-β and CTGF. The two treatments also produced a significant reduction in elastin expression. These results may contribute to explain the favorable effect of laser on soft tissue regeneration without the potential risk of wound-healing defects such as hypertrophic scars or keloids, among others, although *in vivo* studies are warranted to assess the optimal calibration of laser-induced activation to ensure normal scar formation.

Angiogenesis is a complex and decisive physiological process in wound-healing and tissue regeneration^24^, and it depends on VEGF, among other growth factors, and on ECM elements. We achieved a significant increase in VEGF expression with the two-dose treatment, in line with various studies of laser treatments *in vivo* (wound models in rats: 660 nm, 16 mW, 10 J/cm^2^; 660 nm, 30 mW 72 J/cm^2^) and *in vitro* (cultured human fibroblasts: 660 nm, 35 mW, 1–5 J/cm^2^)^25–27^. This rise in VEGF expression would contribute to tissue regeneration.

DDR2 collagen receptors regulate fibroblast proliferation and migration and ECM synthesis, which are crucial in wound-healing. There is also a close relationship between DDR2 and MMP-2, predominant proteases in the ECM that are responsible for wound remodeling. Thus, a decrease in DDR2 was reported to reduce migration and MMP-2 expression in fibroblasts^28^. In the present study, treatment with 940 nm diode laser increased the expression of DDR-2 and MMP-2 in human fibroblasts, consistent with observations^29^ of a significant increase in MMP-2 expression by human fibroblasts treated with other types of laser (erbium: YAG laser, 5 or 10 J/cm^2^).

Myofibroblasts are present in granulation tissue and possess intermediate characteristics between fibroblasts and smooth muscle cells. They play a major role in the inflammation, repair, and remodeling of different tissues. Myofibroblasts are differentiated from fibroblasts and are characterized by the expression of α-actin^30,31^. Both laser treatments of our cultured human fibroblasts produced a significant increase in the expression of the myofibroblast markers α-actin and fibronectin^30^. These results agree with the confocal microscopy observations of intense fibronectin and α-actin expressions after the treatment of fibroblasts treated with laser (940 nm, 0.5 W, 3 or 4 J/cm^2^)^13^. The long-term increase in α-actin gene expression is also associated with higher TGF-β1 expression, which is proposed as a triggering factor for the differentiation from fibroblast to myofibroblast^20^. The results of our study suggest that laser treatment increases the growth and migration of fibroblasts and induces their differentiation, thereby favoring tissue wound healing, seeming the T2 treatment to be a better option in tissue regeneration. However, there have been calls for novel approaches to chronic wounds, including the utilization of laser or platelet-rich plasma^32,33^. These therapies could complement conventional moist wound healing treatments with the aim of reducing the healing time and improving the patient’s quality of life.

In conclusion, the biostimulatory effects of 940 nm diode laser on fibroblasts observed in this study suggest its high therapeutic potential for wound regeneration. However, further animal and human studies are warranted to verify its clinical usefulness.

## Methods

### Cell cultures

The human CCD-1064Sk epithelial fibroblast cell line was purchased from American Type Cultures Collection (ATCC, Manassas, VA, USA) (ATCC: CRL-2076) and maintained in Dulbecco’s Modified Eagle Medium (DMEM; Invitrogen Gibco Cell Culture Products, Carlsbad, CA) with 100 IU/mL penicillin (Lab Roger SA, Barcelona, Spain), 50 μg/mL gentamicin (Braum Medical SA, Jaen, Spain), 2.5 μg/mL amphotericin B (Sigma -Aldrich Co. Chem. Comp., St. Louis, Mo, USA), 1% glutamine (Sigma -Aldrich Co), and 2% HEPES (Sigma -Aldrich Co) supplemented with 10% fetal bovine serum (FBS) (Gibco, Paisley, UK). Cultures were kept at 37 °C in humidified atmosphere of 95% air and 5% CO_2_. Cells were detached from the culture flask with a solution of 0.05% trypsin (Sigma -Aldrich Co) and 0.02% ethylene diamine tetra-acetic acid (EDTA) (Sigma -Aldrich Co) and were then washed and suspended in complete culture medium with 10% FBS.

### Laser irradiation

This study used a diode laser, equipped with a biostimulation hand piece, that operates at a wavelength of 940 nm with maximum power output of 10 W and spot diameter of 400 microns (Biolase Technology, Inc., Irvine, CA).

Cells were seeded at 2 × 10^4^ cells/ml in 24-well plates with an adequate between-well distance to avoid irradiation from proximal wells and scattered irradiation. Cells were then laser-irradiated in uncovered plates at room temperature with 0.5 W power and 4 J/cm^2^ energy density in continuous wave (CW), previously found to be optimal for biostimulatory effect on this cell population^13^, following two treatment guidelines: T1) single energy dose (0 h) and quantitative analysis after 144 h of treatment; T2) two energy doses, one at baseline (0 h) and the other at 72 h, analyzing cells at 72 h after the second dose (144 h after the first dose). A control group of untreated cells was included in all experiments. Before treatments, cells were starved for synchronization by growing them in DMEM without FBS. The irradiation time was determined according to the energy density and power applied as 12.32 seconds.

### RNA extraction and cDNA synthesis (reverse transcription)

The method described by Manzano-Moreno *et al*.^34^ was used to extract the mRNA of cells undergoing T1 or T2 treatment and of control cells cultured under the same conditions. All assays were run in triplicate. Subsequently, an equal amount of RNA (1 μg total RNA in 40 μL total volume) was reverse-transcribed to cDNA and amplified by PCR using the iScript™ cDNA Synthesis Kit (Bio-Rad laboratories, Hercules, CA) in accordance with the manufacturer’s instructions.

### Real-time polymerase chain reaction (q-RT-PCR)

The mRNA of fibroblast growth factor (FGF), connective tissue growth factor (CTGF), vascular endothelial growth factor (VEGF), transforming growth factor β1 (TGF-β1), transforming growth factorβ-receptors (TGFβR1, TGFβR2 and TGFβR3), discoidin domain receptor 2 (DDR2), matrix metalloproteinase-2 (MMP2), α-actin, fibronectin, decorin, and elastin was detected with primers designed using the NCBI-nucleotide library and Primer3-design (Table 2). All primers had been matched to the mRNA sequences of the target genes (NCBI Blast software). Final results were normalized using ubiquitin C (UBC), peptidylprolyl isomerase A (PPIA), and ribosomal protein S13 (RPS13) as stable housekeeping genes^35^.

**Table 2:** Primer sequences for the amplification of cDNA by real-time PCR.

Gene	Sense Primer	Antisense Primer	Amplicon (bp)
FGF	5′-CCCATATTCCCTGCACTTTG-3′	5′-ACCTTGACCTCTCAGCCTCA-3′	195
CTGF	5′-CCTGGTCCAGACCACAGAGT-3′	5′-TGGAGATTTTGGGAGTACGG-3′	194
VEGF	5′-CCTTGCTGCTCTACCTCCAC-3′	5′-CACACAGGATGGCTTGAAGA-3′	197
TGF-β1	5′-TGAACCGGCCTTTCCTGCTTCTCATG-3′	5′-GCGGAAGTCAATGTACAGCTGCCGC-3′	152
TGFβR1	5′-ACTGGCAGCTGTCATTGCTGGACCAG-3′	5′-CTGAGCCAGAACCTGACGTTGTCATATCA-3′	201
TGFβR2	5′-GGCTCAACCACCAGGGCATCCAGATGCT-3′	5′-CTCCCCGAGAGCCTGTCCAGATGCT-3′	139
TGFβR3	5′-ACCGTGATGGGCATTGCGTTTCCA-3′	5′-GTGCTCTGCGTGCTGCCGATGCTGT-3′	173
DDR2	5′-GAACCCAAACATCATCCATC-3′	5′-CTTCATGCCAGAGGCAATTT-3′	199
MMP2	5′-CCAAGAACTTCCGTCTGTCC-3′	5′-TGAACCGGTCCTTGAAGAAG-3′	195
α-actin	5′-TCCTGCTCCTCTCTGTCTCAT-3′	5′-AGTCAGAGCTTTGGCTAGGAA-3′	96
fibronectin	5′-GCCATGACAATGGTGTGAAC-3′	5′-GCAAATGGCACCGAGATATT-3′	200
decorin	5′-GGGCTGGCAGAGCATAAGTA-3′	5′-CAGAGCGCACGTAGACACAT-3′	196
elastin	5′-GGTGTAGGTGGAGCTTTTGC-3′	5′-CTGTTGGGTAACCAGCCTTG-3′	199

Quantitative RT-PCR (q-RT-PCR) was performed using the SsoFast™ EvaGreen® Supermix Kit (Bio-Rad laboratories) in accordance with the manufacturer’s protocol. The comparative Ct method was used for the relative quantification of gene expression. The mRNA concentration for each gene was expressed in ng of mRNA per average ng of housekeeping mRNAs. cDNA of cells from at least three independent cultures were analyzed using qRT-PCR.

### Statistical analysis

R software (R Foundation for Statistical Computing, Vienna, Austria) was used for the statistical analysis. Differences between means were evaluated by Mann-Whitney *U* test. Data are reported as means ± standard deviation (SD). Differences between experimental groups were considered statistically significant at p ≤ 0.05.

## Data Availability

Datasets generated during and/or analyzed during the current study are available from the corresponding author upon reasonable request.

## References

[1] De Donatis A, Ranaldi F, Cirri P (2010). Reciprocal control of cell proliferation and migration. Cell Commun. Signal..

[2] Lemons JMS (2010). Quiescent fibroblasts exhibit high metabolic activity. PLoS Biol..

[3] Gurtner GC, Werner S, Barrandon Y, Longaker MT (2008). Wound repair and regeneration. Nature..

[4] Martin P, Nunan R (2015). Cellular and molecular mechanisms of repair in acute and chronic wound healing. Br J Dermatol..

[5] Rola P, Doroszko A, Derkacz A (2014). The Use of Low-Level Energy Laser Radiation in Basic and Clinical Research. Adv Clin Exp Med..

[6] Bjordal JM, Johnson MI, Iversen V, Aimbire F, Lopes-Martins RAB (2006). Photoradiation in acute pain: A systematic review of possible mechanisms of action and clinical effects in randomized placebo-controlled trials. Acute Pain..

[7] de Paula Eduardo C (2010). Laser phototherapy in the treatment of periodontal disease. A review. Lasers Med Sci..

[8] Machado RS, Viana S, Sbruzzi G (2017). Low-level laser therapy in the treatment of pressure ulcers: systematic review. Lasers Med Sci..

[9] Capon A, Mordon S (2003). Can thermal lasers promote skin wound healing?. Am J Clin Dermatol..

[10] Huertas RM (2014). Effect and clinical implications of the low-energy diode laser on bone cell proliferation. Biol Res Nur.s.

[11] Manzano-Moreno FJ, Medina-Huertas R, Ramos-Torrecillas J, García-Martínez O, Ruiz C (2015). The effect of low-level diode laser therapy on early differentiation of osteoblast via BMP-2/TGF-β1 and its receptors. J Craniomaxillofac Surg..

[12] Medina-Huertas R (2014). The effects of low-level diode laser irradiation on differentiation, antigenic profile, and phagocytic capacity of osteoblast-like cells (MG-63). Lasers Med Sci..

[13] Illescas-Montes R (2017). Cultured Human Fibroblast Biostimulation Using a 940 nm Diode Laser. Materials (Basel)..

[14] Barrientos S, Brem H, Stojadinovic O, Tomic-Canic M (2014). Clinical application of growth factors and cytokines in wound healing. Wound Repair Regen..

[15] Eckes B, Nischt R, Krieg T (2010). Cell-matrix interactions in dermal repair and scarring. Fibrogenesis & Tissue Repair..

[16] Kinoda J (2018). Protective effect of FGF-2 and low-molecular-weight heparin/protamine nanoparticles on radiation-induced healing-impaired wound repair in rats. J. Radiat. Res..

[17] Khoo NK (2014). *In vitro* Therapeutic Effects of Low Level Laser at mRNA Level on the Release of Skin Growth Factors from Fibroblasts in Diabetic Mice. Avicenna J Med Biotechnol..

[18] Damante CA, De Micheli G, Miyagi SPH, Feist IS, Marques MM (2009). Effect of laser phototherapy on the release of fibroblast growth factors by human gingival fibroblasts. Lasers Med Sci..

[19] Klass BR, Grobbelaar AO, Rolfe KJ (2009). Transforming growth factor beta1 signalling, wound healing and repair: a multifunctional cytokine with clinical implications for wound repair, a delicate balance. Postgrad Med J..

[20] Ramos-Torrecillas J, Luna-Bertos ED, Manzano-Moreno FJ, García-Martínez O, Ruiz C (2014). Human fibroblast-like cultures in the presence of platelet-rich plasma as a single growth factor source: clinical implications. Adv Skin Wound Care..

[21] Barrientos S, Stojadinovic O, Golinko MS, Brem H, Tomic-Canic M (2008). Growth factors and cytokines in wound healing. Wound Repair Regen..

[22] Kwan P, Ding J, Tredget EE (2015). MicroRNA 181b Regulates Decorin Production by Dermal Fibroblasts and May Be a Potential Therapy for Hypertrophic Scar. Plos One..

[23] Nirodi CS (2000). Chemokine and chemokine receptor expression in keloid and normal fibroblasts. Wound Repair Regen..

[24] Eming SA, Martin P, Tomic-Canic M (2014). Wound repair and regeneration: Mechanisms, signaling, and translation. Sci Transl Med..

[25] Colombo F (2013). Effect of low-level laser therapy (λ660 nm) on angiogenesis in wound healing: a immunohistochemical study in a rodent model. Braz Dent J..

[26] Fiorio FB (2017). Photobiomodulation therapy action in wound repair skin induced in aged rats old: time course of biomarkers inflammatory and repair. Lasers Med Sci..

[27] Szezerbaty SKF (2018). The effect of low-level laser therapy (660 nm) on the gene expression involved in tissue repair. Lasers Med Sci..

[28] Márquez J, Olaso E (2014). Role of discoidin domain receptor 2 in wound healing. Histo. Histopathol..

[29] Schmitt L (2017). Molecular effects of fractional ablative erbium:YAG laser treatment with multiple stacked pulses on standardized human three-dimensional organotypic skin models. Lasers Med Sci..

[30] Darby IA, Laverdet B, Bonté F, Desmoulière A (2014). Fibroblasts and myofibroblasts in wound healing. Clin Cosmet Investig Dermatol..

[31] Shu DY, Lovicu FJ (2017). Myofibroblast transdifferentiation: The dark force in ocular wound healing and fibrosis. Prog Retin Eye Res..

[32] de Alencar Fonseca Santos J (2018). Effects of Low-Power Light Therapy on the Tissue Repair Process of Chronic Wounds in Diabetic Feet. Photomed Laser Surg.

[33] Ramos-Torrecillas J (2015). Effectiveness of platelet-rich plasma and hyaluronic acid for the treatment and care of pressure ulcers. Biol Res Nur.

[34] Manzano-Moreno FJ (2018). Bisphosphonate modulation of the gene expression of different markers involved in osteoblast physiology: Possible implications in bisphosphonate-related osteonecrosis of the jaw. Int J Med Sci..

[35] Ragni E, Viganò M, Rebulla P, Giordano R, Lazzari L (2013). What is beyond a qRT-PCR study on mesenchymal stem cell differentiation properties: how to choose the most reliable housekeeping genes. J Cell Mol Med..

